# Supplementary material for: Charge-partition pathways in strong-field photoionization of carbonyl sulfide monomers and dimers
Source: Phys Chem Chem Phys. 2026 Sep 7;28(36):22557–66. doi: 10.1039/d6cp02531e (PMC13549219; doi:10.1039/d6cp02531e)
Supplement: Supplementary file 1 [file CP-028-D6CP02531E-s001.pdf]

Supplementary Information (SI) for Physical Chemistry Chemical Physics.
This journal is © the Owner Societies 2026

Supplementary Information

for

Charge-partition pathways in strong-field photoionization of carbonyl sulfide monomers and dimers

Chao He, Xinyue Zhang, Cangtao Yin, Markus Meuwly and Stefan Willitsch

Department of Chemistry, University of Basel, Klingelbergstrasse 80, Basel 4056, Switzerland

Corresponding author: stefan.willitsch@unibas.ch

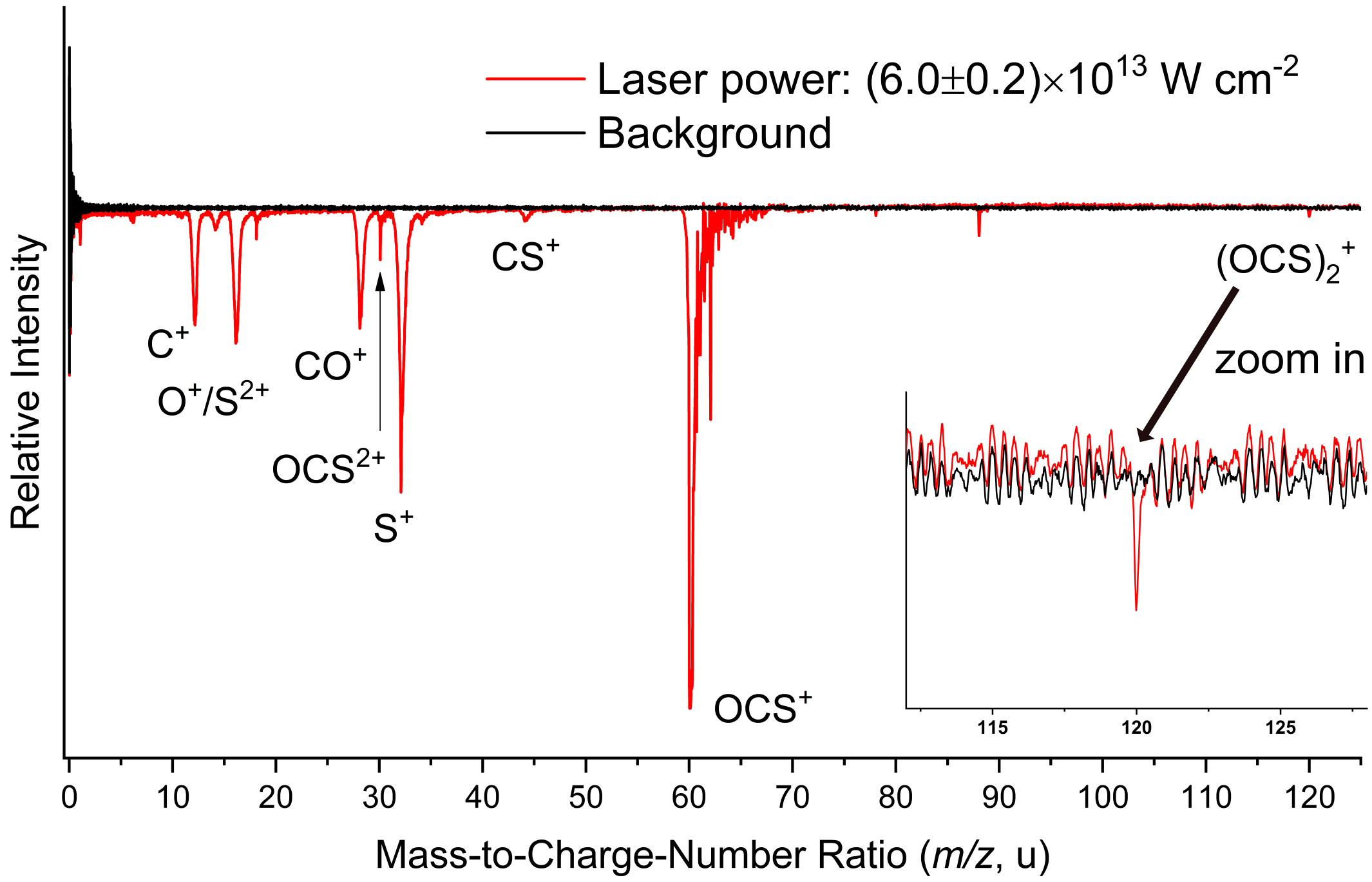

Figure S1: Time-of-flight mass spectra of ionic products generated from 1000 ppm carbonyl sulfide (OCS) seeded in helium (He) by ionization using femtosecond laser pulses (775 nm, 150 fs) at a peak intensity of $(6.0 \pm 0.2) \times 10^{13}$ W, cm^{-2} (red trace). The black trace represents the background signal. The inset highlights the ion signal at m/z = 120 u.

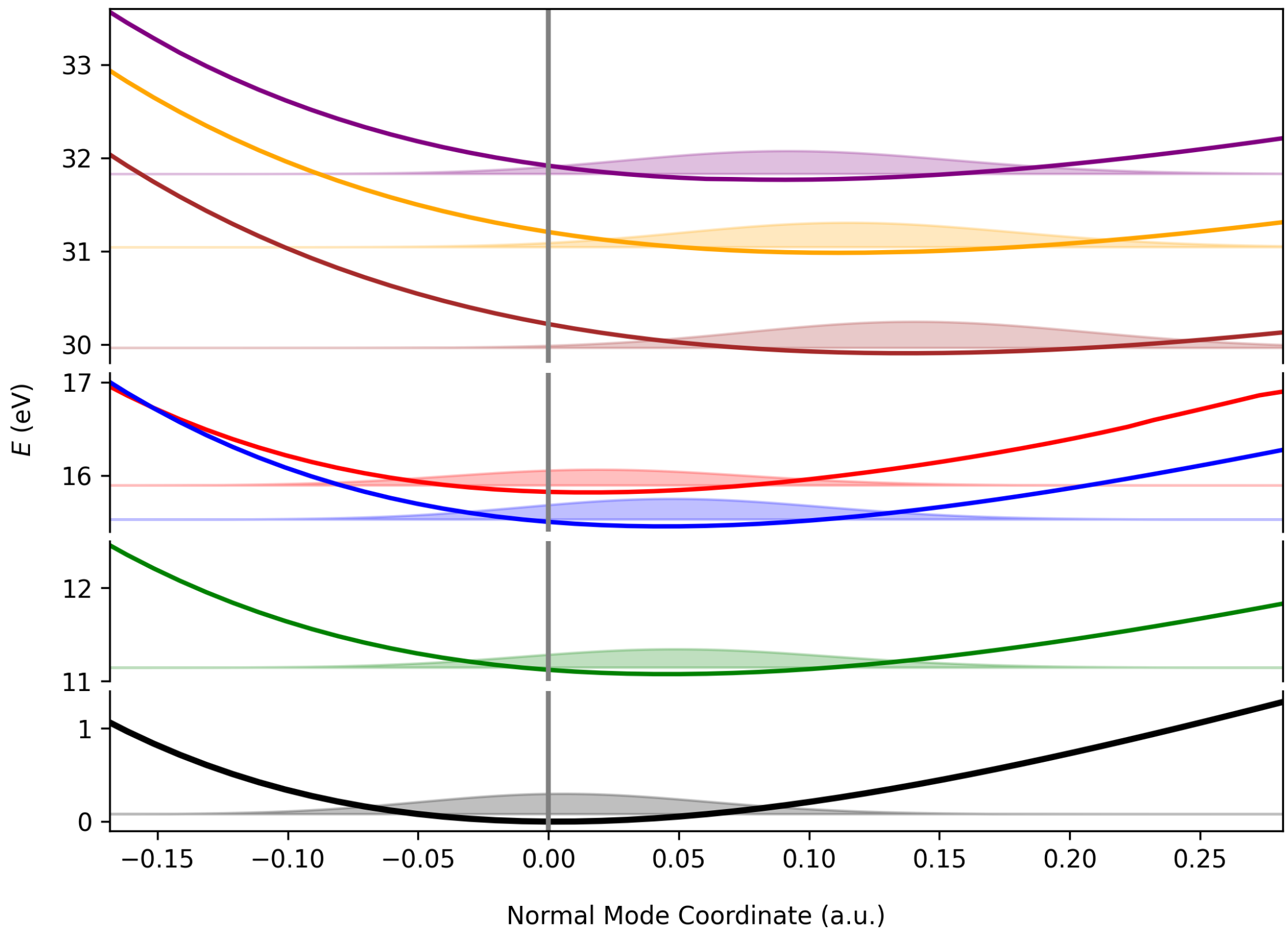

Figure S2: Comparison of potential energy curves of OCS and its cations: neutral ground state (black: $X^1\Sigma^+$), three lowest-lying electronic states of OCS^+ (green: $X^2\Pi$, blue: $A^2\Pi$, red: $B^2\Sigma^+$), three lowest-lying electronic states of OCS^{2+} (brown: $X^3\Sigma^-$, orange: $a^1\Delta$, purple: $b^1\Sigma^+$). The X-axis represents the C-S stretching normal coordinate. The vertical gray line marks the equilibrium structure of neutral OCS. In addition, the ground-state vibrational wavefunctions for each PES are also depicted as shaded areas, colored to match their corresponding PES. The equilibrium geometry of OCS was optimized at the MRCI/aVTZ level with Davidson-relaxed reference correction, while the normal mode vectors were obtained using CCSD(T)/aVTZ.

Table S1: Franck–Condon factors for transitions from the ground vibrational state of neutral OCS in its $X^1\Sigma^+$ ground electronic state to vibrational states v in selected electronic states of OCS^+ and OCS^{2+}.

State	$v = 0$	$v = 1$	$v = 2$	$v = 3$	$v = 4$	$v = 5$	$v = 6$	$v = 7$
OCS^+ ($X^2\Pi$)	0.75	0.22	0.03					
OCS^+ ($A^2\Pi$)	0.75	0.20	0.04	0.01				
OCS^+ ($B^2\Sigma^+$)	0.97	0.03						
OCS^{2+} ($X^3\Sigma^-$)	0.08	0.19	0.24	0.21	0.15	0.08	0.04	0.01
OCS^{2+} ($a^1\Delta$)	0.18	0.29	0.25	0.16	0.08	0.03	0.01	
OCS^{2+} ($b^1\Sigma^+$)	0.33	0.35	0.20	0.08	0.03	0.01		

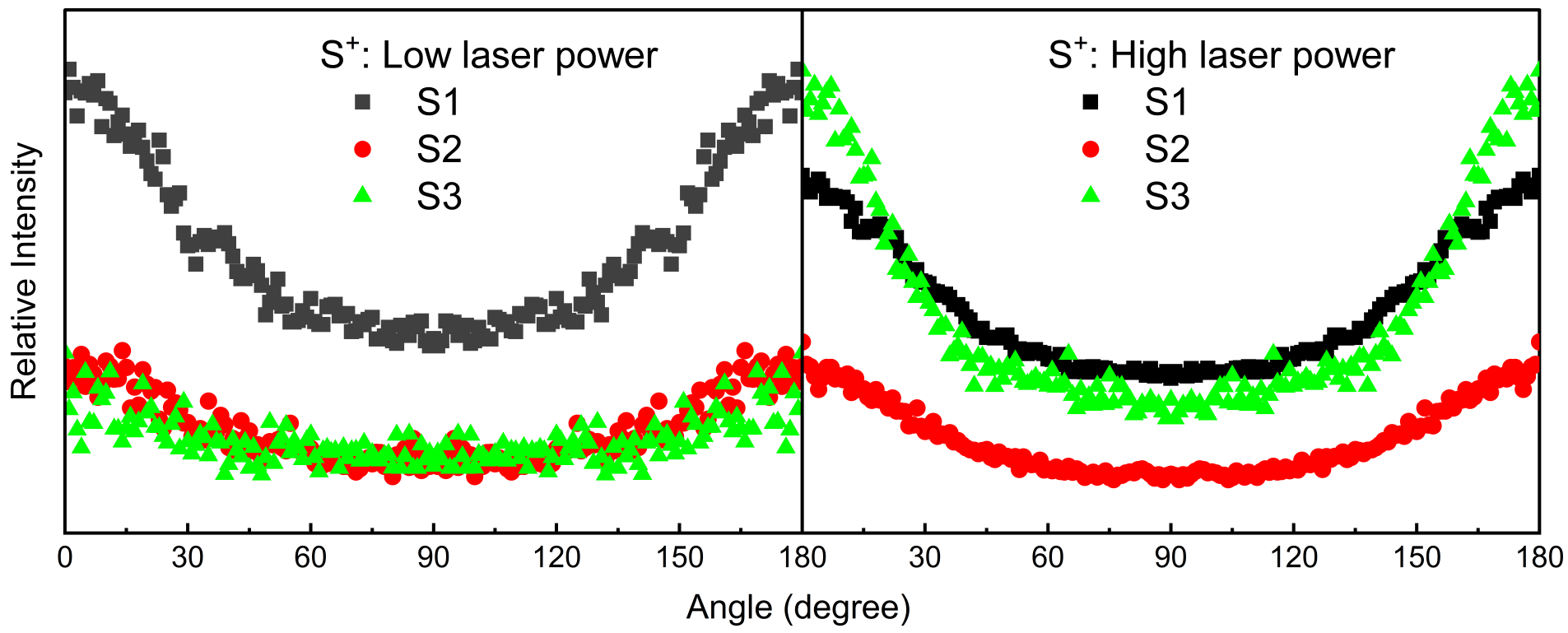

Figure S3: Angular distribution of S1, S2, S3 channels of product S^+ at low (left) and high (right) laser power.

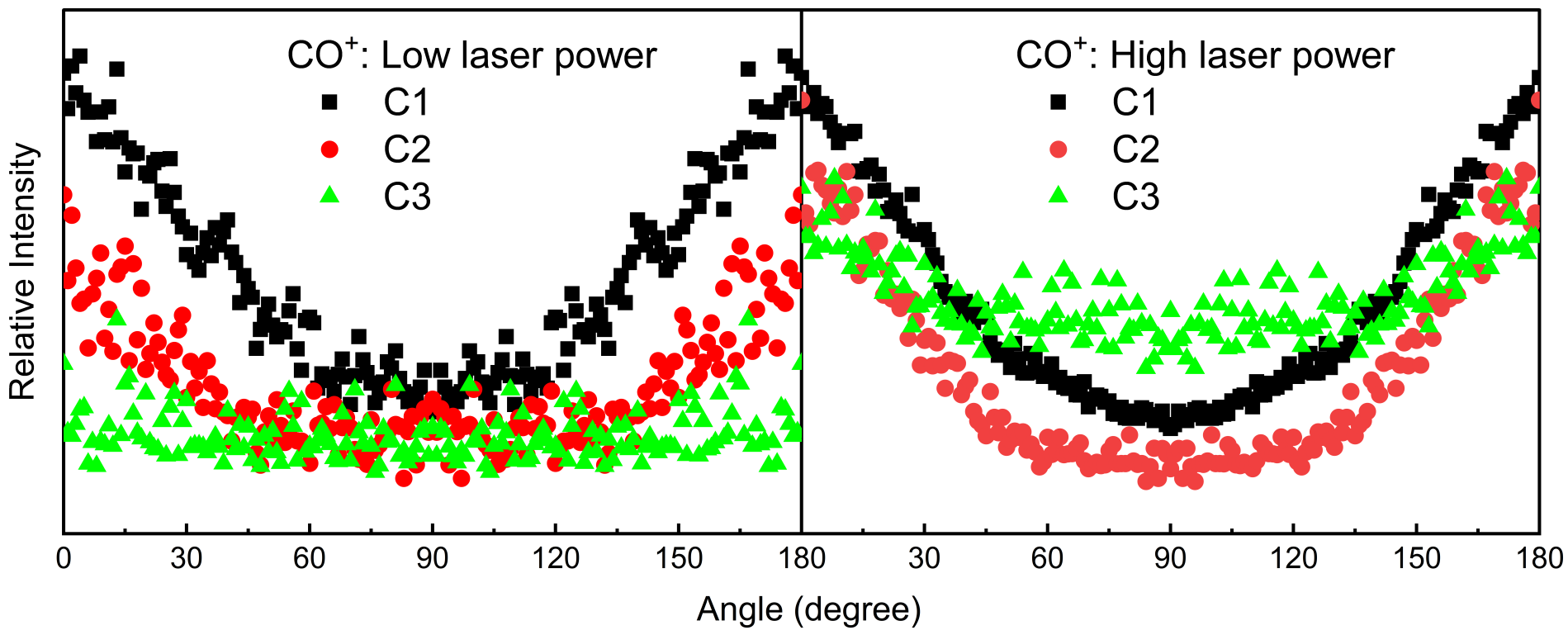

Figure S4: Angular distribution of product channels C1, C2, C3 of CO^+ at low (left) and high laser power (right).
